# Correction to “Validation of the German version of the 25‐item hikikomori questionnaire 25”

**DOI:** 10.1002/mpr.70002

**Published:** 2024-09-13

Hajek, A., Teo, A. R., Zwar, L., & König, H. H. (2024). Validation of the German version of the 25‐item hikikomori questionnaire 25. *International Journal of Methods in Psychiatric Research*, 33(2), e2027.

We noticed that there is an error in the title:

Incorrect title: Validation of the German version of the 25‐item hikikomori questionnaire 25.

Correct title: Validation of the German version of the 25‐item hikikomori questionnaire (HQ‐25).

We apologize for this error.

